# Clinical, Microbiological and Treatment Characteristics of Severe Postoperative Respiratory Infections: An Observational Cohort Study

**DOI:** 10.3390/jpm13101482

**Published:** 2023-10-11

**Authors:** Adela Benítez-Cano, Silvia Bermejo, Sonia Luque, Luisa Sorlí, Jesús Carazo, Irene Zaragoza, Isabel Ramos, Jordi Vallès, Juan P. Horcajada, Ramón Adalia

**Affiliations:** 1 Department of Anesthesiology and Surgical Intensive Care, Hospital del Mar, Parc de Salut Mar, 08003 Barcelona, Spain; sbermejo@psmar.cat (S.B.); jcarazo@psmar.cat (J.C.); irene.zaragoza.garcia@psmar.cat (I.Z.); mramos@psmar.cat (I.R.); jvalles@psmar.cat (J.V.); radalia@psmar.cat (R.A.); 2 Infectious Pathology and Antimicrobial Research Group (IPAR), Institut Hospital del Mar d’Investigacions Mèdiques (IMIM), 08003 Barcelona, Spain; sluque@psmar.cat (S.L.); lsorli@psmar.cat (L.S.); jhorcajada@psmar.cat (J.P.H.); 3 Pharmacy Department, Hospital del Mar, Parc de Salut Mar, 08003 Barcelona, Spain; 4 Infectious Diseases Department, Hospital del Mar, Parc de Salut Mar, 08003 Barcelona, Spain; 5 CIBERINFEC, ISCIII-CIBER de Enfermedades Infecciosas, Instituto de Salud Carlos III, Av. de Monforte de Lemos, 5, 28029 Madrid, Spain

**Keywords:** postoperative, severe respiratory infection, pneumonia, tracheobronchitis, critically ill

## Abstract

Respiratory infections are frequent and life-threatening complications of surgery. This study aimed to evaluate the clinical, microbiological and treatment characteristics of severe postoperative pneumonia (POP) and tracheobronchitis (POT) in a large series of patients. This single-center, prospective observational cohort study included patients with POP or POT requiring intensive care unit admission in the past 10 years. We recorded demographic, clinical, microbiological and therapeutic data. A total of 207 patients were included, and 152 (73%) were men. The mean (SD) age was 70 (13) years and the mean (SD) ARISCAT score was 46 (19). Ventilator-associated pneumonia was reported in 21 patients (10%), hospital-acquired pneumonia was reported in 132 (64%) and tracheobronchitis was reported in 54 (26%). The mean (SD) number of days from surgery to POP/POT diagnosis was 6 (4). The mean (SD) SOFA score was 5 (3). Respiratory microbiological sampling was performed in 201 patients (97%). A total of 177 organisms were cultured in 130 (63%) patients, with a high proportion of Gram-negative and multi-drug resistant (MDR) bacteria (20%). The most common empirical antibiotic therapy was a triple-drug regimen covering MDR Gram-negative bacteria and MRSA. In conclusion, surgical patients are a high-risk population with a high proportion of early onset severe POP/POT and nosocomial bacteria isolation.

## 1. Introduction

Nosocomial pneumonia remains a significant cause of morbidity and mortality, with an attributable mortality rate ranging from 30 to 50% [1,2]. Up to 50% of nosocomial pneumonia cases are postoperative and are thus termed postoperative pneumonia (POP). This category includes pneumonia acquired during the postoperative period, whether related or unrelated to mechanical ventilation (MV), termed ventilator-associated pneumonia (VAP) [3,4]. 

Despite advances in both surgical and anesthetic techniques, POP and postoperative tracheobronchitis (POT) remain prevalent conditions that are often associated with high morbidity and mortality rates. POP is the third leading cause of postoperative infectious complications, following urinary tract infection and surgical site infection, with an incidence of between 9% and 40% and associated mortality rates of 20% and 45% [3,5,6,7,8,9]. 

The characteristics of surgical patients predispose them to the development of respiratory infections in the postoperative setting. Proposed risk factors for POP in this population include antibiotic prophylaxis, general anesthesia, the administration of neuromuscular blocking agents, MV during the intervention and difficulty in the management of respiratory secretions due to pain or the presence of drains [10,11].

The pathogenesis of POP and POT may be multifactorial, involving several factors such as the colonization of the gastrointestinal tract, the aspiration of contaminated secretions and compromised host defenses (due to critical illness, surgical insult, comorbidities and medications). These infections are mostly bacterial and often polymicrobial, especially in patients at a high risk of bronchoaspiration [4]. 

Despite their high prevalence and clinical relevance, POP and POT remain poorly studied clinical entities with a scarcity of published research, which is mainly focused on specific types of surgery [4,12,13,14]. In this scenario, the main objective of this study was to evaluate the clinical and microbiological characteristics and the therapeutic management of POP and POT requiring admission to the intensive care unit (ICU).

## 2. Material and Methods

This single-center, retrospective observational cohort study was conducted at a tertiary ICU at Hospital del Mar in Barcelona, Spain, between January 2013 and January 2023. Ethics approval was obtained from the research ethics committee, CEIC Parc de Salut Mar (approval number 2023/11061). The need for written consent was waived, as the study consisted of a secondary analysis of existing data. 

This study was performed in accordance with the STROBE statement guidelines for reporting observational studies [15].

### 2.1. Patient Enrolment

The inclusion criteria were as follows: age ≥ 18 years, diagnosis of severe POP or POT: nosocomial pneumonia or severe tracheobronchitis diagnosed within the first 15 days after surgery [12] and requiring admission to the ICU. 

The exclusion criteria consisted of patients younger than 18 years, a previous diagnosis or suspicion of prior lung infection before surgery, a diagnosis of pneumonia or tracheobronchitis within the first 48 h after surgery (community-acquired pneumonia or tracheobronchitis), pulmonary infection diagnosed more than 15 days after the intervention and patients not requiring admission to the ICU.

In patients with recurrent episodes of respiratory infection during the same hospital admission, only the first episode was included. 

### 2.2. Data Collection

The following data were collected on the day of surgery: demographics, ASA score [16], ARISCAT score [17], type of intervention (emergency, scheduled), surgical specialty (abdominal, thoracic, urological, neck, vascular, traumatology, neurosurgery), surgical incision site (open, laparoscopic, peripheral), surgical antibiotic prophylaxis (type of antibiotic, days of antibiotic prophylaxis), anesthetic technique (general, regional) and duration of intraoperative mechanical ventilation. The data recorded at ICU admission were severity at the time of the pneumonia diagnosis (SOFA score [18]), the presence of sepsis or septic shock [19]), the type of pneumonia (hospital-acquired pneumonia, VAP, hospital-acquired tracheobronchitis, ventilator-associated tracheobronchitis), data about the patient’s immune profile (C-reactive protein, pro-calcitonin and white blood cell count) and oxygenation (PaO_2_/FiO_2_). We also recorded the presence of risk factors for multidrug-resistant (MDR) [20] pathogens (prior intravenous antibiotic use within 90 days and 5 or more days of hospitalization prior to the occurrence of pneumonia) [21], days from surgery to diagnosis and type of ICU ventilation (invasive or non-invasive (MV/NIMV)).

The data recorded on microbiological diagnosis consisted of the microbiological sampling technique (invasive versus non-invasive) and microbiological sampling type (sputum, tracheal aspirations, bronchial aspirations, protected distal specimen, bronchoalveolar lavage).

Microbiological data comprised the type of infection (monomicrobial, polymicrobial), isolated microorganisms and antibiotic susceptibility (minimum inhibitory concentration).

Data on therapeutic management included initial empirical therapy and the appropriateness of initial empirical treatment according to the isolated microorganism.

Data on outcomes consisted of the length of ICU and hospital stay and 30-day, ICU and in-hospital mortality. 

### 2.3. Definitions and Data Collection

The diagnosis of pneumonia was based on standard clinical and laboratory criteria and was defined as a new or progressive radiological pulmonary infiltrate plus two or more of the following characteristics: temperature > 38 °C or <35 °C, leucocyte count > 11,000 or <4000 cells/mm^3^ or purulent respiratory secretions [21]. POP was considered if the infection was diagnosed between the third and fifteenth day after an elective or emergency surgical intervention.

In patients without a radiological pulmonary infiltrate, an episode of tracheobronchitis was considered if it met at least two of the following criteria [22]: fever ≥ 38 °C, leucocytosis > 11,000 cells/μL or leukopenia < 4000 cells/μL, the presence of purulent bronchial secretions and at least one of the following microbiological criteria: positive quantitative culture of lower respiratory tract samples with minimal contamination (e.g., bronchoalveolar lavage (BAL) ≥ 10^4^ CFU/mL) or a positive quantitative culture of endotracheal aspirate (ETA) samples (e.g., ETA ≥ 10^5^ CFU/mL). An episode of POT was considered for inclusion if it met the previous criteria and required intravenous antibiotic treatment and ICU admission.

Hospital-acquired pneumonia (HAP) and hospital-acquired tracheobronchitis (HAT) were defined as pneumonia or tracheobronchitis not incubating at the time of hospital admission and occurring ≥ 48 h after admission. VAP and ventilator-associated tracheobronchitis (VAT) were defined as pneumonia or tracheobronchitis occurring ≥48 h after endotracheal intubation [21]. 

POP and POT were considered early onset if they were diagnosed <5 days after surgery and late onset if they were diagnosed after the 5th day after surgery. 

An MDR pathogen is defined as non-susceptible to at least one agent in three or more antimicrobial categories [20]. 

Empirical antibiotic treatment was defined as appropriate if the infecting microorganism was found to be susceptible in vitro to the drug administered [23]. 

ICU and 30-day mortality was considered as death from any cause during the ICU admission or in the 30 days following the POP or POT diagnosis, and in-hospital mortality was defined as death from any cause occurring during the hospital stay.

### 2.4. Diagnostic Management

The procedures followed for the diagnosis and treatment of the included patients were based on the recommendations of the guidelines for the management of HAP/VAP of the European Respiratory Society (ERS), European Society of Intensive Care Medicine (ESICM), European Society of Clinical Microbiology and Infectious Diseases (ESCMID) and Asociación Latinoamericana del Tórax (ALAT) [1] and the Clinical Practice Guidelines of the Infectious Diseases Society of America and the American Thoracic Society [21].

The methods used for microbiological diagnosis (blood cultures, respiratory samples) were determined by the attending physicians based on the procedures usually applied in the ICU. If there were no contraindications, clinicians from the Pulmonary Department obtained an invasive respiratory sample through fiberoptic bronchoscopy. Whenever possible, respiratory samples were obtained prior to the start of antibiotic treatment [1,21].

### 2.5. Microbiology

The organisms isolated were identified according to the microbiological procedures routinely used in the microbiological laboratory. Antibiotic susceptibility testing of the isolated pathogens was determined using the Vitek2^®^ automated system (Biomerieux, Craponne, France) and was interpreted according to EUCAST breakpoints (European Committee on Antimicrobial Susceptibility Testing) [24].

For each sample collected, we collected the results of the direct examination, culture and bacterial identification.

### 2.6. Statistical Analysis

Categorical variables are expressed as absolute frequencies and percentages and quantitative variables as means and standard deviations. The comparison of continuous variables was performed using the Student *t*-test for variables with a normal distribution and using the nonparametric Mann–Whitney U test when normality could not be assumed. For dichotomous variables, the chi-square and the Fisher exact tests were applied. Logistic regression was used to explore various risk factors associated with the primary endpoint of the study. Univariate analyses were performed separately for each of the risk factor variables to ascertain the odds ratio and 95% confidence interval (CI). The Cox proportional hazards regression model was used to perform multivariate analyses of 30-day all-cause mortality, and the results were reported as the hazard ratio (HR) and 95% confidence interval (CI). Variables with a *p* value ≤ 0.2 in the univariate analysis and clinically relevant variables were included in the multivariate models. All *p*-values were two-tailed, and the statistical significance was < 0.05. The SPSS (Statistical Package for the Social Sciences) version 18.0 statistical package was used throughout. 

## 3. Results

### 3.1. Demographics and Patient Baseline Characteristics

During the study period, 207 patients were included, of which 152 (73%) were men. The mean (SD) age was 70 (13) years, and the median (SD) ARISCAT score was 46 (19). 

The demographic and clinical characteristics of the included patients are summarized in Table 1. 

Surgical antibiotic prophylaxis was administered in all patients for a maximum duration of 48 h, mostly as beta-lactam antibiotic monotherapy. At the time of POP/POT diagnosis, 149 patients (69%) were already receiving antibiotic treatment for a different infectious focus (mostly abdominal infections). 

The mean (SD) number of days from surgery until POP/POT diagnosis was 6 (4) days. A total of 106 patients (51%) had early POP/POT (<5 days from surgery). The mean (SD) SOFA score at POP/POT diagnosis was 5 (3).

A total of 153 patients were diagnosed with some type of POP (HAP (64%) and VAP (10%) patients). The remaining patients (26%) had tracheobronchitis (VAT (10%) and HAT (4%)). During ICU admission, 101 patients (49%) required invasive MV and 37 (18%) required NIVM. Forty-eight (36%) HAP and twenty-three (51%) HAT patients required MV after POP diagnosis.

### 3.2. Microbiological Diagnostic Data

Microbiological sampling was performed in 201 patients (97%), including bronchoscopic techniques in 127 (61%). 

A wide variety of methods were used. A non-invasive sampling technique was applied in 74 patients (36%): sputum in 30 (14.5%) and endotracheal aspirate in 44 (21%). An invasive sampling technique was applied in 127 patients (61%): protected distal specimen in 59 (28.5%), bronchial aspirations in 63 (30%) and bronchoalveolar lavage in 5 (2%). In 6 patients (3%), no microbiological sample was available.

### 3.3. Microbiological Results

A total of 177 organisms were cultured from pulmonary samples in 130 patients (63%). 

The pulmonary infection was monomicrobial in 84 patients (41%) and polymicrobial in 46 patients (22%), and no microorganism was isolated in 77 patients (37%).

The organisms cultured from respiratory samples are presented in Table 2. 

A total of 19 (11%) Gram-negative MDR pathogens were found. The most frequent MDR pathogens isolated were MDR *P. aeruginosa* (16 isolates (9%)) and *Extended-expectrum betalactamase-producer (ESBL) Enterobacterales* (14 isolates (8%)). All MDR except one (an ESBL-*Escherichia coli)* were isolated in patients with risk factors for MDR pathogens. 

### 3.4. Antibiotic Treatment

The most common empirical antibiotic therapy was a triple-drug regimen, accounting for 86 patients (41%). This regimen often consisted of a carbapenem plus an aminoglycoside and nebulized polymyxin (specifically, nebulized sodium colistimethate (CMS)) or a carbapenem plus colistin for MDR Gram-negative bacteria cover plus linezolid for MRSA cover.

The initial treatment regimens are summarized in Table 3.

The initial empirical antibiotic treatment was considered appropriate in 174 patients (83%).

### 3.5. Outcomes

The SOFA score at the time of POP/POT diagnosis was positively correlated with the length of the ICU stay (Spearman’s rho 0.145, *p* = 0.05). A longer ICU stay was also observed in patients with late onset POP/POT compared to those with early onset POP/POT (30.85 (29.18) versus 22.87 (26.75); *p* = 0.02) and in patients requiring MV during ICU admission compared to those without MV (31.20 (27.99) versus 21.7 (21.18); *p* = 0.012).

No differences in mortality were observed between different types of POP/POT or between POP and POT. The thirty-day mortality was higher in patients with septic shock than in those without septic shock (68.8% versus 31.3%; *p* = 0.008). A tendency to a higher 30-day mortality among patients with late onset POP/POT was observed compared to those with early onset POP/POT (33.9% versus 23.0%; *p* = 0.071).

The multivariate analysis found that only age was a significant predictor for 30-day all-cause mortality (age > 75 years (HR 3.176; 95% CI, 1.674–6.026; *p* = 0.00)). VM ≥ 3 days (HR 1.768; 95% CI, 0.913–3.424; *p* = 0.09) and SOFA score at the time of POP/POT diagnosis (HR 1.095; 95% CI, 0.991–1.209; *p* = 0.07) showed a tendency for predicting mortality.

## 4. Discussion

We present a cohort of 207 critically ill patients with postoperative nosocomial pneumonia or tracheobronchitis during the first fortnight after an elective or emergency surgical intervention and requiring ICU admission. As far as we know, this is the first study on severe POP or POT and is one the few studies on nosocomial pneumonia in this population.

Although the timeline from surgery to POP/POT diagnosis is not clearly defined, we used the same definition as Montravers et al. [12], although other definitions have been previously used [14,17,25]. We believe that POP/POT occurring within this chosen time window can reasonably be attributed to the surgical intervention. Respiratory infections diagnosed during the first 48 h of admission are probably the result of previous infection (community-acquired pneumonia), while nosocomial lung infections occurring later during hospital admission are more likely to be related to prolonged hospital or ICU stay.

In the present study, the mean number of days from surgery to POP/POT diagnosis was 6 (4) days, indicating that approximately half of the patients had early onset POP/POT (<5 days from surgery). Our results are in accordance with those of previous studies [7,12,26] reporting a high incidence of early onset POP.

Surgical patients are considered to be at a high risk of developing respiratory complications. Some predictive models have been created to stratify patient risk. In our study, the ARISCAT (Assess Respiratory Risk in Surgical Patients in Catalonia risk-score) was calculated before surgery [17]. This risk index is based on seven independent risk factors (low preoperative arterial oxygen saturation, acute respiratory infection during the previous month, age, preoperative anemia, upper abdominal or intrathoracic surgery, surgical duration of at least 2 h and emergency surgery). The presence of one or more of these factors allows for the identification of patients at risk of developing postoperative complications. The mean (SD) ARISCAT score in our patients was 46 (19), reflecting their high risk of developing pulmonary complications. Identifying patients at risk of developing respiratory infective complications would allow for the adoption of prevention measures or at least enhanced postoperative vigilance.

Almost all patients (96%) in our cohort underwent surgery with general anesthesia, thus requiring intraoperative MV. Specific risk factors that have been repeatedly related to the development of postoperative pulmonary complications are the intraoperative administration of neuromuscular blocking agents, difficulty in the management of respiratory secretions due to pain or the presence of drains and certain surgical sites (upper abdominal, open thoracotomy) [17,25,27,28]. However, other potential risk factors have not been well studied, such as the need for intubation and MV during the intervention.

No differences in the percentage of POP/POT were found between emergency and scheduled surgery. The most frequent surgical specialty was abdominal surgery, followed by thoracic surgery, and most of the patients underwent open surgery. Risk factors for the development of POP in surgical patients are irritation of the diaphragm, traction of the chest wall, inhibition of the respiratory and cough reflex center, postoperative incision pain and long-term bed rest. These factors are especially relevant in open surgery. Indeed, laparoscopic procedures have been reported to decrease the risk of POP by 50% compared with open surgery [14,29]. In our hospital, surgery changed substantially over the study period, with an increase in the number of laparoscopic procedures (either abdominal or thoracic), especially during the last few years. However, the number of patients who developed POP after a laparoscopic procedure remained low (15%) in our cohort.

In the present study, POP was unrelated to MV (HAP) in 64% of the patients, while VAP occurred in only 10%. Whereas the incidence of VAP has gradually decreased in the last few years, probably related to preventive measures [30,31], the incidence of HAP remains high, ranging from 5 to 20 cases/1000 hospital admissions [32]. In our group, more than one-third of patients with HAP required MV during admission. Although VAP-associated mortality has traditionally been considered to be higher than HAP-related mortality, recent evidence shows that mortality is higher in HAP requiring MV, thus reflecting the poor clinical progression of the pneumonia [33]. In one-third of patients, tracheobronchitis was diagnosed (VAT or HAT). Interestingly, half of the patients with HAT required MV during ICU admission, probably reflecting progression to a more severe entity. VAT has been described in studies on nosocomial respiratory tract infections in the ICU since the 1990s [34]. While several studies suggest that VAT is associated with an increased duration of MV and the length of the ICU stay [22,35,36], information on HAT remains scarce due to the heterogeneity of definitions with nonspecific and subjective signs. As far as we know, this is the first study including HAT in a cohort of critically ill surgical patients. We believe that patients with clear signs of respiratory infection and positive respiratory culture should be managed as POP, especially if they require ICU admission.

In our cohort, an invasive distal respiratory sample was obtained through a bronchoscospic method for a high proportion of patients (61%), thus allowing for a more precise identification of the causative organisms and susceptibility patterns. The use of non-invasive diagnostic methods (e.g., sputum and endotracheal aspirate collection) could lead to the over-identification of bacteria by the initial direct examination of samples [37]. Consequently, the latest European guidelines suggest obtaining distal quantitative samples to reduce antibiotic exposure [1].

Some studies have shown that pulmonary infections in surgical patients have certain microbiological characteristics, with a high prevalence of Gram-negative and nosocomial pathogens, even in patients without risk factors [7,12]. In our study, a large proportion (85%) of Gram-negative nosocomial pathogens (*Enterobacterales* and *Pseudomonas* spp.) were found, even in early onset POP in patients without risk factors for MDR bacterial infection. Few studies have investigated the microbiological features of POP [12,38,39]. Our results show similarities with those of previous studies [12], although the proportion of Gram-positive bacteria in our patients was lower than in previous reports. The administration of antibiotic prophylaxis in surgical patients has been proposed as an explanation for the differences between surgical and non-surgical nosocomial pneumonia. Another factor that could be related to our results is that most of our patients were on antibiotics at the time of POP diagnosis (mostly for abdominal infections requiring emergency surgery).

The wide range of empirical therapies prescribed in our patients could, at least partially, be explained by microbiological variability and changes in antibiotic policies during the last few years based on the continuous evaluation of our data. Consequently, we believe that any statement could be made regarding antibiotic therapy. Further studies are required to clarify the optimal antibiotic treatment and to define appropriate guidelines in this setting.

More than one-fifth of patients died during ICU admission, almost one-third of them during hospital admission. The most recent data have indicated an attributable mortality of 13% for VAP [2], with a mortality rate of up to 69% in surgical patients. Although the authors of that study did not explain the differences in mortality, they hypothesized that it could be related to differences in the severity of illness and comorbidity in surgical patients compared with medical and trauma patients. In addition, POP has been identified as an independent risk factor for overall and disease-specific survival in some types of cancer patients after surgical resection [13,40]. It has been postulated that complex postoperative recovery processes like POP may have deleterious effects in cancer recovery and progression through effects on the patients’ general health status, as well as through the inhibition of the immune response to tumor cell proliferation, leading to lower disease-specific survival.

Three significant predictors for 30-day all-cause mortality were identified in the multivariate analysis: age > 75 years, (HR 3.176; 95% CI, 1.674–6.026; *p* = 0.00), ≥3 days of MV (HR 1.768; 95% CI, 0.913–3.424; *p* = 0.09) and SOFA score at the time of POP/POT diagnosis (HR 1.095; 95% CI, 0.991–1.209; *p* = 0.07), although only the age showed statistical significance. These findings are more likely to be secondary to severity than to the specific characteristics of POP/POT. However, as POP/POT-attributable mortality was not investigated, no conclusions can be drawn.

Our study has several limitations. First, its observational nature does not allow comparisons to be made between our results and the results of randomized controlled trials. However, we believe that the data reported here provide a comprehensive view of the difficulties encountered by clinicians in the management of these high-risk patients. We did not record, and therefore could not analyze, data related to intraoperative factors that have been associated with POP, such as colloids and blood transfusions. Second, we only included patients with POP requiring ICU admission and therefore lack information about less severe forms of pneumonia. Third, it is difficult to estimate the impact of POP/POT on prognosis, as some patients with POP/POT probably had other complications impacting their outcomes.

In conclusion, our study suggests that surgical patients are a high-risk population. A large proportion of patients with severe POP/POT had early onset infection with a notable presence of nosocomial bacteria, even in the early phase. There is an urgent need for vigilance and prevention measures in this susceptible population.

## Figures and Tables

**Table 1:** Patients’ characteristics and clinical data.

Demographic and Clinical Data	Total (N = 207)
Male, n (%)	152 (73)
Age (years), mean (SD)	70 (13)
ASA score, n (%)	
ASA score I or II	33 (16)
ASA score III	123 (59)
ASA score IV	51 (25)
ARISCAT score, mean (SD)	46 (19)
Type of surgery, n (%)	
Emergency surgery	102 (49)
Elective surgery	105 (51)
Surgical specialty, n (%)	
Abdominal surgery	108 (52)
Thoracic surgery	32 (16)
Orthopedic and spine surgery	18 (9)
Vascular surgery	16 (8)
Neurosurgery	12 (6)
Urological surgery	10 (5)
Head and neck surgery	3 (1)
Others	8 (4)
Surgical incision site	
Open upper abdominal	97 (44)
Peripheral	40 (19)
Laparoscopy	25 (12)
Open thoracotomy	16 (8)
Head and neck	16 (8)
Thoracoscopy	7 (3)
Open lower abdominal	6 (3)
Type of anesthesia, n (%)	
General	198 (96)
Regional	9 (4)
Intraoperative mechanical ventilation (minutes), mean (SD)	250 (133)
Other clinical data	
Type of pneumonia, n (%)	
Hospital-acquired pneumonia	132 (64)
Hospital-acquired tracheobronchitis	45 (22)
Ventilator-associated pneumonia	21 (10)
Ventilator-associated tracheobronchitis	9 (4)
SOFA score, mean (SD) ª	5 (3)
Septic shock, n (%) ª	80 (39)
Sepsis, n (%) ª	127 (61)
Immune profile	
C-reactive protein (mg/dL), mean (SD)	20 (14)
Pro-calcitonin (ng/mL), mean (SD)	12 (28)
White blood cell count (×10^3^/mm^3^), mean (SD)	14,584 (7614)
PaO_2_/FiO_2_, mean (SD)	234 (100)
Mechanical ventilation, n (%)	
Invasive mechanical ventilation	101 (49)
Non-invasive mechanical ventilation	37 (18)
Outcomes	
ICU stay (days), mean (SD)	26 (25)
Hospital stay (days), mean (SD)	48 (36)
ICU mortality, n (%)	40 (22)
30-day mortality, n (%)	51 (25)
In-hospital mortality, n (%)	69 (29)

ª At the time of POP diagnosis; ASA score, American Society of Anesthesiologists Classification score [16]; ARISCAT score, Assess Respiratory Risk in Surgical Patients in Catalonia [17]; SD, standard deviation; SOFA score, Sepsis-related Organ Failure Assessment score [18]; ICU, intensive care unit.

**Table 2:** Microorganisms isolated from respiratory samples.

	Total n, (%)	Non-Risk Factors MDR n, (%)	Risk Factors MDR n, (%)
Gram-negative bacteria			
*Pseudomonas* spp.	25 (14)	4 (16)	21 (14)
MDR *Pseudomonas aeruginosa*	16 (9)	0	16 (10)
*Escherichia coli*	14 (8)	1 (4)	13 (9)
*Serratia* spp.	10 (6)	1 (4)	9 (6)
*Klebsiella* spp.	14 (8)	6 (24)	8 (5)
ESBL-producer *Enterobacterales*	14 (8)	1 (4)	13 (9)
Carbapenemase-producing *Enterobacterales*	1 (1)	0	1 (1)
Other *Enterobacterales*	16 (9)	3 (12)	13 (9)
*Stenotrophomona maltophilia*	12 (7)	0	12 (8)
*Haemophilus* spp.	10 (6)	5 (20)	5 (3)
*Acinetobacter* spp.	4 (2)	0	4 (3)
MDR *Acinetobacter baumannii*	1 (1)	0	1 (1)
Miscellaneous	11 (6)	1 (4)	10 (7)
Gram-positive bacteria			
*Staphylococcus aureus*	12 (7)	2 (8)	10 (7)
*Streptococcus pneumoniae*	4 (2)	1 (4)	3 (2)
*Methicillin-resistant S. aureus*	3 (2)	0	3 (2)
Other *streptococci*	2 (1)	0	2 (1)
Miscellaneous	1 (1)	0	1 (1)
Fungi			
*Aspergillus fumigatus*	3 (2)	0	3 (2)
*Cryptococcus neoformans*	1 (1)	0	1 (1)
Viruses		0	
*Herpes simplex virus*	2 (1)	0	2 (1)
*Cytomegalovirus*	1 (1)	0	1 (1)

ESBL, extended-spectrum beta-lactamase; MDR, multi-drug resistant; MDR, multi-drug resistant bacteria.

**Table 3:** Initial empiric antibiotic treatment.

Empirical Therapy	Patients n, (%)
Monotherapy	49 (24)
β-lactam antibiotic	21 (10)
Carbapenem	16 (8)
Fluoroquinolones	3 (2)
Other	9 (4)
Double combination	72 (35)
Carbapenem + polymyxin *	23 (11)
Carbapenem + oxazolidinone (Linezolid)	13 (6)
β-lactam antibiotic + fluoroquinolones	8 (4)
Carbapenem + glycopeptides	5 (2)
β-lactam antibiotic + aminoglycoside	3 (2)
Carbapenem + aminoglycoside	2 (1)
Carbapenem + fluoroquinolones	1 (1)
Other combinations	17 (8)
Triple-drug therapy	86 (42)
Carbapenem + aminoglycosides + polymyxin **	26 (13)
Carbapenem + oxazolidinone (Linezolid) + polymyxin **	20 (10)
Carbapenem + oxazolidinone (Linezolid) + fluoroquinolones	5 (2)
Miscellaneous	35 (17)

* 22 patients with carbapenem + nebulized colistin and 1 patient with carbapenem + iv + nebulized colistin (sodium colistimetathe). ** nebulized colistin (sodium colistimetathe).

## Data Availability

The datasets generated and/or analyzed during the current study are available from the corresponding author upon reasonable request.
